# *Ganoderma lucidum* Extract Modulates Gene Expression Profiles Associated with Antioxidant Defense, Cytoprotection, and Senescence in Human Dermal Fibroblasts: Investigation of Quantitative Gene Expression by qPCR

**DOI:** 10.3390/cimb47020130

**Published:** 2025-02-18

**Authors:** Harald Kühnel, Markus Seiler, Barbara Feldhofer, Atefeh Ebrahimian, Michael Maurer

**Affiliations:** Department of Applied Life Sciences, Bioengineering, University of Applied Sciences Campus Wien, Favoritenstraße 222, 1100 Vienna, Austriabarbara.feldhofer@fh-campuswien.ac.at (B.F.);

**Keywords:** *Ganoderma lucidum*, cellular senescence, senolytic, senomorphic, etoposide, human dermal fibroblast

## Abstract

Cellular senescence plays a crucial role in skin aging, with senescent dermal fibroblasts contributing to reduced skin elasticity and increased inflammation. This study investigated the potential of *Ganoderma lucidum* (Reishi) ethanol extract to modulate the senescent phenotype of human dermal fibroblasts. Reishi powder of two different vendors was used. The extract was produced by extracting the Reishi powder for at least three weeks in 40% ethanol at room temperature. Etoposide-induced senescent fibroblasts were treated with Reishi extracts from two commercial sources for 14 days. Gene expression analysis was performed using qPCR to assess senescence makers, antioxidant defense, and extracellular matrix remodeling. Results showed that Reishi extracts significantly upregulated antioxidant and cytoprotective genes, including Heme oxygenase 1 (HO-1), γ-Glutamylcysteine synthetase (γGCS-L), and NAD(P)H dehydrogenase [quinone] 1 (NQO1), compared to untreated controls. Importantly, Reishi treatment suppressed the expression of p16^INK4a^, a key marker of cellular senescence, while transiently upregulating p21^Cip1^. The extracts also demonstrated potential senolytic properties, reducing the percentage of senescent cells as measured by senescence-associated β-galactosidase staining. However, Reishi treatment did not mitigate the upregulation of MMP1 and IL-8 in one Reishi treatment group, indicating differences in the preparations of different vendors. These findings suggest that *Ganoderma lucidum* extract may help alleviate some aspects of cellular senescence in dermal fibroblasts, primarily through enhanced antioxidant defense and cytoprotection, potentially offering a novel approach to combat skin aging.

## 1. Introduction

Cellular senescence is a complex biological process characterized by a stable cell cycle arrest accompanied by distinct phenotypic alterations [1,2]. This phenomenon plays crucial roles in various physiological and pathological processes, including embryonic development, tissue repair, tumor suppression, and aging. Senescent cells undergo significant changes in gene expression, metabolism, and secretory profile, with the latter known as the senescence-associated secretory phenotype (SASP) [3].

Dermal fibroblasts are mesenchymal cells that reside in the dermis, the layer of skin beneath the epidermis. These cells play critical roles in maintaining skin homeostasis, synthesizing and remodeling the extracellular matrix (ECM), and participating in wound-healing processes. Dermal fibroblasts produce various ECM components, including collagens, elastin, and proteoglycans, which are essential for skin structure and function [4]. In the context of cellular senescence, dermal fibroblasts are of particular interest due to their involvement in skin aging and age-related skin disorders. As these cells undergo senescence, they exhibit characteristic changes such as enlarged and flattened morphology, increased expression of senescence-associated β-galactosidase (SA-β-gal), and activation of the cyclin-dependent kinase inhibitor p16^INK4a^ and p21^CiP1^ [5,6]. Senescent dermal fibroblasts contribute to skin aging through various mechanisms. They show reduced proliferative capacity and altered ECM production, leading to decreased skin elasticity and wrinkle formation. Additionally, the SASP of senescent fibroblasts includes pro-inflammatory cytokines, matrix metalloproteinases, and growth factors that can further modify the skin microenvironment and influence neighboring cells [4,7].

Etoposide-induced senescence in fibroblast cells serves as a well-established model for studying cellular senescence. This process involves using etoposide, a topoisomerase II inhibitor, which induces DNA damage, particularly double-strand breaks. When administered at appropriate concentrations to fibroblasts, etoposide triggers a senescence response rather than apoptosis [8,9]. The characteristics of etoposide-induced senescence in fibroblasts include a stable cell cycle arrest, typically in the G1 phase, accompanied by distinct morphological changes, as well as other hallmarks of senescence [10,11]. Etoposide-induced senescent fibroblasts develop an SASP, characterized by the secretion of various factors, including pro-inflammatory cytokines, growth factors, and matrix-degrading enzymes. However, the specific SASP profile can vary depending on the fibroblast type and experimental conditions.

This model of senescence in fibroblasts has significant applications in research. It provides a valuable tool for studying the mechanisms of cellular senescence, DNA damage responses, and the impact of senescent cells on tissue microenvironments [12]. Furthermore, it allows for the exploration of potential interventions to modulate senescence. One of the key advantages of using etoposide to induce senescence is its ability to generate a relatively homogeneous population of senescent cells within a short timeframe, allowing for more controlled and reproducible experiments compared to replicative senescence models.

Importantly, this model also provides insights into the relationship between DNA damage, oxidative stress, and senescence, as the DNA damage response triggered by etoposide can lead to increased reactive oxygen species (ROS) production, further exacerbating cellular senescence. Etoposide treatment reliably induces DNA damage-related senescence in human articular chondrocytes, evidenced by the loss of proliferative capacity, DNA damage accumulation, and the expression of some SASP components [13].

Oxidative stress and ROS play a crucial role in the induction and maintenance of cellular senescence in dermal fibroblasts. Studies have shown a significant increase in ROS levels in aged human fibroblasts in vitro and in aged rat skin in vivo, indicating that ROS accumulation is a key regulator of the aging process in the dermis. This excessive ROS production is often associated with mitochondrial dysfunction in fibroblasts, creating a vicious cycle where mitochondrial-derived ROS activates the mammalian target of rapamycin complex 1 (mTORC1), leading to further mitochondrial dysfunction and ROS production [14].

The nuclear factor erythroid 2-related factor 2 (Nrf2) pathway, which regulates antioxidant responses, is reduced during fibroblasts’ photoaging and chronological aging [14,15]. The enhancement of Nrf2 signaling can attenuate aging and inflammation in dermal fibroblasts. Senescent fibroblasts can also induce senescence in neighboring cells through paracrine signaling, creating a self-perpetuating cycle of senescence in the dermis [14]. Oxidative stress, caused by an imbalance between ROS production and elimination, is a key driver of cellular senescence and aging [16,17], but targeting senescent cells can extend the lifespan and health span [17].

Nevertheless, using the whole plant extract preserves the natural balance of these components, potentially leading to enhanced overall efficacy due to synergistic interactions between compounds as well as a broader spectrum of biological activities. Whole plant extracts are often classified as dietary supplements or traditional herbal medicines, which typically face less stringent regulatory requirements compared to isolated compounds.

On the one hand, this study is intended to show that plant extracts that are already used on humans are able to have a senolytic effect and thus represent a possibility to influence the aging process. On the other hand, fibroblasts could become senescent as collateral damage of chemotherapy with etoposide. The induced senescence could negatively influence tumor growth by releasing SASP factors to neighboring cells and thus result in a negative prognosis. It may be possible to alleviate these effects with these plant extracts.

Given these findings, *Ganoderma lucidum* extract shows promise as a potential modulator of cellular senescence and oxidative stress in dermal fibroblasts. To investigate this further, we conducted a series of experiments to evaluate the effects of Reishi extract on etoposide-induced senescence in human dermal fibroblasts (HDFs), focusing on gene expression profiles associated with antioxidant defense, cytoprotection, and senescence markers.

## 2. Materials and Methods

### 2.1. Definition of Reagents

#### 2.1.1. Preparation of Extract and Standard Substances

Reishi fruiting bodies powders from two suppliers, Sunday Natural (Sunday Natural Products GmbH; Potsdamer Straße 83; 10785 Berlin, Germany) and Nature’s Finest (Nutrisslim, Ljubljana, Slovenija), were dissolved in 40% ethanol, which was prepared with pure water and 99.9% ethanol (Australco, Spillern, Austria) to a concentration of 100 mg/mL, respectively. Thereafter, the mixtures were stored in 250 mL Duran^®^ Schott bottles (VWR, Radnor, PA, USA) in the dark at room temperature for three to four weeks to let them fully extract the potent substances to the liquid phase. After that period, the extractions were filtrated by means of a pleated filter (MN 615 1/4, Macherey-Nagel^®^, Düren, Germany) and stored in a refrigerator at 4 °C until use. Overall, three extracts of both Reishi powders were prepared during all experiments. In the preparation of the second and third ones, the Schott bottles were additionally shaken two times per week in the extraction phase to enhance the process of extraction. Vitamin C, as a stock solution, was prepared by dissolving 20 mg of the respective powder in 10 mL 40% ethanol. The preparations were stored in the refrigerator at 4 °C until use. For the preparation of media for cell culture treatment, amounts of 500 µL of the 40% extract or the control were diluted in 19.5 mL of the media, resulting in a 1% ethanol solution.

#### 2.1.2. Dry Matter Determination

Empty glass eprouvettes were heated and weighed until weight constancy before they were each filled with 2 mL of the supernatant or final Reishi extract and incubated for at least three days at 70–80 °C. When all the water–ethanol liquid was evaporated, the remaining solid was cooled in a desiccator and subsequently also weighed until weight constancy. Because the weight of the empty eprouvettes after heating them in a sterilization cabinet hardly changed in the first three dry matter determinations, this step was skipped for the following measurements. All measurements were performed at least in duplicate.

#### 2.1.3. DPPH Assay

First, a DPPH stock solution was prepared by dissolving 394 mg of DPPH in 10 mL 99.9% methanol (100 mM) by means of an ultra-wave bath. The received solution was diluted 1:100 freshly before every new analysis. Thereafter, 100 µL of the to-be-analyzed extract or solution was pipetted in a transparent 96-well plate (Greiner Bio-One, Kremsmünster, Oberösterreich, Austria) and diluted 11 times in 1:2 steps with its corresponding medium (ethanol or water). This step was completed for the control, and 100 µL of either 40% ethanol or pure water was added into the wells as blanks. Subsequently, the same amount of the 1:100 DPPH solution was pipetted to each filled well except for the controls, to which 40% ethanol or pure water was used. For better mixing, the plates were shaken for 20 s in the TECAN Spark^®^ Multimode Microplate Reader before incubating at room temperature for half an hour in the dark. Next, the absorbance of each well was measured (TECAN, Männedorf, Switzerland) at a wavelength of 517 nm, and the radical scavenging activity was calculated from them, using Equation (1):

(1) rsa [%] = (1 − (A − C)/B) × 100

A: absorbance of the measured sample;

C: control (absorbance of samples with ethanol or water instead of DPPH);

B: blank (ethanol or water instead of the samples).

### 2.2. Cell Culture

#### 2.2.1. Medium Preparation

First, 11.996 g/L of Gibco™ DMEM/F-12 powder (Thermo Fisher Scientific, Waltham, MA, USA, 02451) and 2.438 g/L NaHCO_3_ were dissolved in pure water with the help of a magnetic stirrer. Then, 1% (of the total volume) of thawed Gibco™ Pen Strep (Thermo Fisher Scientific, Waltham, MA, USA, 02451, Cat. No. 15-140-122) was added to the mixture while stirring and, together with 10% SAFC^®^ FBS (Sigma Aldrich, St. Louis, MO, USA; Burlington, MA, USA, Cat. No. 12003C), it was sterile-filtrated with a bottle-top filter. Before use, the FBS was heat-sterilized in a water-bath for half an hour at 56 °C.

#### 2.2.2. Cells

HDFs (ATCC) were thawed at a passage number of seven and cultivated in Greiner Bio-One (Kremsmünster, Oberösterreich, Austria) T-flasks (Cellstar^®^ TC) in DMEM medium. At passage number of 13, a working cell bank was established by aliquoting and freezing the cells in liquid nitrogen at −196 °C. When cells were needed for an experiment, individual vials were thawed and passaged to the desired number of cells. To create a growth curve, cells were passaged continuously until passage 26.

#### 2.2.3. Presto Blue Assay and SA-ß-Gal Assay

The PrestoBlue™ assay and SA-ß-Gal assay were performed as described in Imb et al. [11]; data of this publication were used for presenting the senolytic effect. Young cells were seeded in 24-well plates (3500 cells/cm^2^, 1000 µL medium per well). The Reishi extract was diluted 1:3 in 40% ethanol and 37.5 µL of this extract was added in 962.5 µL medium in the 24-well plate. The extract treatment was performed for 48 h.

Presto Blue staining was performed on day three: 500 µL of the old medium was removed, 500 µL of fresh medium was put back in, and 50 µL of Presto Blue per well was added; the samples were placed in the incubator for 30 min, and then, 100 µL of each was added to a black 96-well plate and the fluorescence was measured. Presto Blue does not harm the cells, so the same 24-well plates were stained for SA-ß-Gal the day after (day 4). Treatments represented the young treatment group.

The old treatment group was terminated as follows: The young cells were seeded in 24-well plates (3500 cells/cm^2^, 1000 µL medium per well). The next day, the cells were treated with etoposide for 48 h. After the removal of the etoposide, the cells were incubated 12 d with regular medium changes. Then, the extract treatment was started the same way as for the young treatment group on day 14 after the etoposide treatment: Presto Blue staining was performed and, thereafter, a medium change was performed and, on the next day, the cells were stained for SA–ß-Gal.

#### 2.2.4. Phospho-Histone H2A.X Assay

This was performed according to Kühnel et al. [10]; briefly, the cells were seeded in chamber slides for 16 h and incubated in 50 µM etoposide for two days. Blood-derived products were added for one day (FCS, CPRP, and HAS); as a control, the cells were incubated in normal growth medium without damaging treatment. Briefly, the cells were fixed in 4% formaldehyde in PBS for 15 min at room temperature. The fixative was aspirated and the cells were rinsed three times in PBS for 5 min. Blocking was performed for 60 min with PBS supplemented with 5% normal serum and 0.3% Triton™ X-100 (Sigma Aldrich, St. Louis, MO, USA; Burlington, MA, USA). After aspiration of the blocking agent, the primary antibody Phospho-Histone H2A.X (Ser139) Antibody (#2577 Cell Signaling, Danvers, MA, USA) was added overnight at 4 °C. After rinsing three times in PBS for 5 min, the specimens were incubated in fluorochrome-conjugated secondary antibody diluted in goat-anti-rabbit polyclonal Fab fragment Ab labeled with AF-488 (3 µg/mL, Jackson Laboratories, Bar Harbor, MN, USA) antibody dilution buffer (1% BSA in PBS, 0.3% Triton™ X-100) for 1 h at room temperature in the dark and then rinsed in PBS as above. The nuclei were stained with DAPI (Sigma Aldrich, St. Louis, MO, USA; Burlington, MA, USA, D9542) 1:1000 in antibody dilution buffer 10 min at 37 °C. The slides were washed with PBS, and mounting was achieved by adding 20 µL Fluoromount-GTM (Southern Biotechnology, Thermo Fisher Scientific, Waltham, MA, USA, 02451); they were covered with the high-precision coverslip No. 1.5H.

#### 2.2.5. Senescence Model

HDFs were treated with 50 µM etoposide in 75 cm^2^ T-flasks, and, after two days, the medium was changed to regular DMEM growth medium, but the cells were still incubated at 37 °C for three to four weeks to let them become fully senescent. During this period, the medium was changed twice a week. In the end, the cells were harvested by treating them with 2 mL trypsin-EDTA for around 20 min. They were centrifuged, and RNA was extracted from the received cell pellet.

#### 2.2.6. Main Experiment

For the main cell culture experiment performed in duplicate, two different Reishi extracts were prepared in each of the two experiments. The cells were seeded in a density of 3500 cells per cm^2^ in 20 mL DMEM growth medium. The next day, they were treated with 25 µM etoposide, and after two days of incubation, the media were changed for the respective group-specific media (the two different Reishi types, the ethanol control, and the control without treatment). The day after and twice a week for the following two weeks, cells from one flask of each group were harvested for qPCR. In addition, 10 mL of the cells were stored at −80 °C for further analyses, and the group-specific medium was changed in the flasks. Also, cells were counted in the Countess Cell Counter after harvesting them, right before their RNA was extracted. The group-specific media were prepared in freshly autoclaved Duran^®^ Schott bottles. Microscopic observations: For observing the morphological changes of the cells throughout the cell culture experiments and for microscopic pictures, a camera (Olympus, Shinjuku, Tokyo, Japan) attached to the transmitted light microscope was used.

#### 2.2.7. IL-6 ELISA

For the measurement of the IL-6 content in the cell-conditioned media, an ELISA kit (Human IL-6 CytoSet™ from Invitrogen™, Carlsbad, CA, USA) was used. An anti-human IL-6 antibody (0.125 mg/0.125 mL) was used as the coating antibody. For detection, an anti-human IL-6 biotin antibody (0.25 mg/0.125 mL) was employed, with streptavidin–HRP facilitating the detection. Recombinant human IL-6 was used for calibration. The IL-6 ELISA was carried out following the supplier’s instructions.

### 2.3. qPCR-Analysis

#### 2.3.1. RNA Extraction

After the harvesting of the cells, their RNA was extracted using the RNeasy Mini Kit from (QIAGEN, Hilden, Germany). First, the samples were centrifuged, and their supernatant was discarded. Then, the received cell pellet was dissolved in 300 or 650 µL RLT buffer containing 10 mol DTT (Roth, Karlsruhe, Germany), depending on the number of cells used. A DTT stock solution was prepared, which was then used for all RNA extractions. Subsequently, the same amount of 70% ethanol was added to every mixture, and after homogenizing by pipetting, the mixtures were centrifuged for 15 s at 8 g. The flowthrough from this and the following three steps was discarded. Thereafter, 700 µL of wash buffer was added to each spin column and centrifuged for 15 s. Next, 500 µL RLT buffer was added to the columns twice; the spin column was centrifuged for 15 s after the first addition and for two minutes after the second addition. Afterward, the spin columns were dried in new collection tubes while centrifuging for one minute at max speed. Last, the RNA was eluted in a new smaller collection tube with 40 µL of pure water by centrifuging at 8 g for one minute and then immediately frozen to −80 °C if not subsequently synthesized to cDNA.

#### 2.3.2. Measurement of RNA and DNA

The Invitrogen™ Qubit™ RNA BR Assay Kit (Thermo Fisher Scientific, Waltham, MA, USA, 02451) was used to measure the concentration of the extracted RNA. First, a working solution was prepared by diluting the Qubit™ RNA BR Reagent 1:200 with its RNA BR Buffer. Then, 2–10 µL of each sample was added to 198–190 µL (total 200 µL) of the freshly prepared working solution, as well as 10 µL of each of the two standards to 190 µL of the working solution. These were vortexed for four seconds, and, after a two-minute incubation at room temperature, the concentration of RNA was measured with the Qubit 2.0 fluorometer.

#### 2.3.3. cDNA-Synthesis

The measured RNA concentration was used to calculate the RNA sample volume + additional nuclease-free water (Roth) for a final concentration of 100 ng or 1 µg per 15 or 20 µL reaction volume. The remaining 5 µL comprised 1 µL iScript™ Reverse Transcriptase (Bio-Rad, Hercules, CA, USA) and 4 µL 5x iScript Reaction Mix. After mixing all substances in 0.2 mL PCR SingleCap reaction tubes (Biozym, Hessisch Oldendorf, Germany), the samples were incubated in the MJ Research PTC-200 Gradient Thermal Cycler (lids heated) according to Table 1.

#### 2.3.4. Core Procedure

##### Primer Preparation

Before the bought primer pairs (Microsynth, Balgach, Switzerland) were used for the qPCR, they were centrifuged for a few seconds, and the stated amount of nuclease-free water for reaching 100 µM was pipetted to the respective forward and reverse primers. Then, the primer pairs were diluted in 1.5 mL Eppendorf tubes^®^ (Hamburg, Germany) to 1:10 (per individual primer) and 1:100 (primer pair) as the final concentrations for the qPCR analyses and stored at −20 °C until use.

##### qPCR Execution and Evaluation

The reaction volume of each PCR 0.1 mL Tube (Biozym, Hessisch Oldendorf, Germany) was 10 µL, consisting of 5 µL iTaq Universal SYBR^®^ Green Supermix (Bio-Rad, Hercules, CA, USA), 3 µL of the respective 1:100 diluted primer pair, and 2 µL of the respective diluted cDNA sample. For most of the analyses, a master mix was created with SYBR^®^ Green and the diluted primer pair, following which the cDNA sample was added last to the qPCR tubes. After all tubes were filled, they were closed and transferred to the Corbett (QIAGEN) Research Rotor-Gene 6000 Real-Time PCR machine for amplification and detection (Table 2). All operations until this step were performed on ice. The AriaMx Real-Time PCR System was utilized for a few analyses due to a defective Rotor-Gene 6000 monitor, but the main experiments were all carried out on the Rotor-Gene 6000 (Eppendorf, Hamburg, Germany).

The Melt Curve Analysis was ramped from 65 °C to 95 °C in 0.5 °C steps of five seconds each. Both the qPCR melt and quantitation curves were created with the Rotor-Gene 6000 software. The threshold of the quantitation curves was always set to 0.02.

#### 2.3.5. Screening of Genes

##### Comparison of Gene Expression Between Young and Old Cells

The primers for many genes associated with oxidative stress, the skin matrix, and aging in general, as well as for suitable reference genes for HDFs, were searched in the literature (Table 3) and tested for their gene expression in not only the young but also the senescent cells with qPCR in duplicates. The formula used was according to Equation (2):

(2) fold change = 2^(−∆∆Ct)

∆∆Ct =〖∆Ct〗_(treated sample) − 〖∆Ct〗_(untreated sample)

〖∆Ct〗_(treated sample) = 〖Ct〗_(GOI treated) − 〖Ct〗_(ref gene treated)

〖∆Ct〗_(untreated sample) = 〖Ct〗_(untreated GOI) − 〖Ct〗_(untreated ref.gene)

GOI: gene of interest

**Table 3:** Genes and primers for qPCR experiments. Bold letters represent housekeeping genes.

Gene Name	Forward Sequence (5′-3′)	Reverse Sequence (5′-3′)	**Ref.**
***TUBA1A1***	**CTTCGTCTCCGCCATCAG**	**CGTGTTCCAGGCAGTAGAGC**	[18]
***VAMP7***	**CAAACATGCTTGGTGTGGAG**	**AAATTAAAGGCTCGGGAACG**	[18]
*TMEM199*	CACCAGCATCTGAGAGAAAGG	CCGTGGAGGCTTCACAAC	[18]
*L3MBTL2*	CCAAGACCAAGAGGTTCTGC	TTTGGTCGGTGGTTTTCC	[18]
*NRF2*	CGGTATGCAACAGGACATTG	GTTGGGGTCTTCTGTGGAGA	[19]
*KEAP1*	CACAGCAATGAACACCATCC	TGTGACCATCATAGCCTCCA	[19]
*BACH1*	TGTGCTTAGAGAAGGATGCTGCTC	TCTTCGTTTCTTCAGGTTCCATTGC	[19]
*HO-1 1*	GAGACGGCTTCAAGCTG	GTGTGTAGGGGATGACC	[19]
*HO-1 2*	GAGGAGTTGCAGGAGCTGCT	GAGTGTAAGGACCCATCGGA	[20]
*FTL*	TCTCGGCCATCTCCTGCTTCTG	CGCCTTCCAGAGCCACATCATC	[19]
*FTH*	GCCGCCGCCTCTCCTTAGTC	CAGTTTCTCAGCATGTTCCCTCTCC	[19]
*NQO1*	CGGCTTTGAAGAAGAAAGG	CTCGGCAGGATACTGAA	[19]
*γ GCS-L*	TCACCTCCTATTGAAGATGG	GGTTACTATTTGGTTTTACCTGT	[19]
*γ GCS-H*	GCAGAGGAGTACACCC	CCACTTCCATGTTTTCAAGG	[19]
*TXNRD1*	CCTATGTCGCTTTGGAG	CCCTACGGTTTCTAAGCC	[19]
*TXN*	CTGCTTTTCAGGAAGCCTTG	ACCCACCTTTTGTCCCTTCT	[19]
*GSHPx*	GGCTACTCTCTCGTTTCCTTTC	GTTCTTGGCGTTCTCCTACAG	[19]
*SOD1*	AGTGCAGGGCATCATCAATTTCGAGCAG	GATGCAATGGTCTCCTGAGAGTGAGATC	[19]
*SOD2 1*	GTCACCGAGGAGAAGTACCAGGAG	CACCAACAGATGCAGCCGTCAG	[19]
*CAT*	CATTCGATCTCACCAAGGTTTGGCC	AGCACGGTAGGGACAGTTCACAGG	[19]
*SESN1-T1*	GGCAAACCATTTTGAGGAAA	TGGTCCCTGTCCTAGTGGTC	[19]
*SESN1-T2*	GCTGGGCTGCAAGCAGTG	CCAAGTTCCTCGTCCTGGT	[19]
*SESN2*	GCACCTACACCCCCTAGTGA	GTCTTCCACAAAGCACAGCA	[19]
*SESN3*	AGTGCTGCGGAAGGATAAAA	CCATGCGCAACATGTAAAAC	[19]
*Col1A1 1*	AGACATCCCACCAATCACCTG	GGCAGTTCTTGGTCTCGTCAC	[21]
*Col1A1 2*	AAGGGACACAGAGGTTTCAGTGG	CAGCACCAGTAGCACCATCATTTC	[22]
*ELN*	GCCCCTGGATAAAAGACTCC	GTCCTCCTGCTCCTGCTGT	[23]
*MMP1*	AGTGACTGGGAAACCAGATGCTGA	CTCTTGGCAAATCTGGCCTGTAA	[24]
*MMP3*	CTGGACTCCGACACTCTGGA	CAGGAAAGGTTCTGAAGTGACC	[24]
*CISD2*	TCCCAGTCCCTGAAAGCATT	ACGAACTGCAAGGTAGCCAAGA	[25]
*SIRT1*	AGCCTTGTCAGATAAGGAAGGA	ACAGCTTCACAGTCAACTTTGT	[26]
*TERT*	CACCTGCCGTCTTCACTTCC	GTGAACAATGGCGAATCTGG	[27]
*GDF11 1*	CCACCACCGAGACCGTCATT	GAGGGCTGCCATCTGTCTGT	[28]
*GDF11 2*	GCAAACTGCGGCTCAAGG	GCTAATGACGGTCTCGGTGG	[29]
*FOXO1*	TCATGTCAACCTATGGCAG	CATGGTGCTTACCGTGTG	[26]
*LMNB1 1*	AAGCAGCTGGAGTGGTTGTT	TTGGATGCTCTTGGGGTT	[30]
*LMNB1 2*	GGGAAGTTTATTCGCTTGAAGA	ATCTCCCAGCCTCCCATT	[30]
*JAK2*	TCTGGGGAGTATGTTGCAGAA	AGACATGGTTGGGTGGATACC	[31]
*MICA*	TAAAATCCGGCGTAGTCCTG	GCATGTCACGGTAATGTTGC	[32]
*PD-L1*	TGGCATTTGCTGAACGCATTT	TGCAGCCAGGTCTAATTGTTTT	[33]
*ULBP1*	CCTGGAGCCTTCTCATCATC	AGGCCTTGAACTTCACACCA	[32]
*ULBP2*	CGCTACCAAGATCCTTCTGTG	GGGATGACGGTGATGTCATA	[32]
*ULBP4*	GACCTCAGGATGCTCCTTTG	GTGCACCGTTCTGCTTCAC	[32]
*MT1X*	GCTCCTGTGCCTGTGCCG	AGCAAACGGGTCGGGTTGTAC	[19]
*MT1E*	GCCCGACCTCCGTCTATAA	AACAAGCAGTCAGGCAGTTG	[19]
*MT2A*	CGCCGCCGGTGACTCCTG	ACGGTCACGGTCAGGGTTGTAC	[19]
*MT1G*	TCCTGTGCCGCTGGTGTCTC	ACGGGTCACTCTATTTGTACTTGGG	[19]
*IL-1β*	GGACAGGATATGGAGCAACAAGTGG	TCATCTTTCAACACGCAGGACAGG	[34]
*IL-6*	GACAGCCACTCACCTCTTCAGAAC	GCCTCTTTGCTGCTTTCACACATG	[34]
*TNF-α*	AAGGACACCATGAGCACTGAAAGC	AGGAAGGAGAAGAGGCTGAGGAAC	[34]
*hCOQ10A*	TTTCAAGGATGCTGGCTCTT	GGCCTCAGCTTGTCAAATTC	[35]
*MSRA*	TGGTTTTGCAGGAGGCTATAC	GTAGATGGCCGAGCGGTACT	[19]

##### Further Evaluation Through Standard Curves

Of most of the genes, especially those with a high difference in expression and rather low Ct values, the primers were tested further for their efficiency and standard curve linearity. Standard curves were created by making a dilution series of at least nine 1:10 steps with nuclease-free water (Roth) in 1.5 or 2.0 mL Eppendorf tubes. Also, special attention has been paid to their linear range since the Ct values of the treated and untreated samples had to lie in that zone for their eligibility. The formula for the calculation of the efficiencies was according to Equation (3):

(3) E = (〖10〗^(−1/slope) − 1) × 100

E: the efficiency in %;

Slope: the gradient of the calibration curve.

#### 2.3.6. Main qPCR Experiment 

The RNA samples were synthesized to cDNA at a concentration of 1 µg per 20 µL reaction volume. Thereafter, they were diluted to 1:30 and aliquoted before they were frozen at −80 °C for the following qPCR analyses.

The formula for calculating the relative gene expression of every tested gene in the final main experiment was according to Equation (4):

(4) relative gene expression =〖RQ〗_GOI/(geom.mean[RQ]_(ref.genes)

〖RQ〗_GOI = (E + 1)^(∆Ct GOI)

[RQ]_(ref.genes) = (E + 1)^(∆Ct ref.gene)

∆Ct GOI = average Ct control − 〖Ct〗_GOI

∆Ct ref.gene = average 〖Ct〗_control − 〖Ct〗_(ref.gene)

RQ: relative quantities;

GOI: gene of interest;

E: efficiency of the standard curve.

### 2.4. Statistics

All analyses were performed using Prism 10 for Windows 64-bit Version 10.4.0 (621) on 23 October 2024. The normality was checked using the Shapiro–Wilk test, and differences between groups were calculated by simple ANOVA. Linear and non-linear regressions were also performed. The non-linear regression for ELISA was performed by variable slope (four parameters).

## 3. Results

### 3.1. Definition of Reagents

#### 3.1.1. Fruiting Bodies Extracts

Fruiting bodies extracts of Reishi were prepared as described in Section 2.1.1 with powder from two commercial online suppliers: Sunday Natural (Sunday Natural Products GmbH; Potsdamer Straße 83; 10785 Berlin, Germany) and Nature’s Finest (Nutrisslim d.o.o; Obrtniška ulica 4, 1292 Ig, Slovenija).

**Figure 1:**
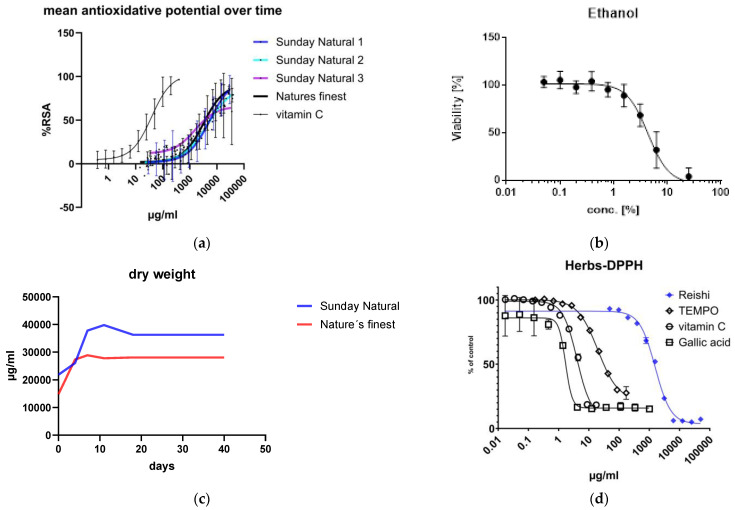
Definition of fruiting bodies extracts (Reishi, *Ganoderma lucidum*): (**a**) comparative mean antioxidative potential of different Reishi extracts over time. Standard deviations were quite high but comparable to those of the vitamin C standard, indicating the poor reproducibility of the DPPH assay. Values are expressed as percent radical scavenging activity (%RSA). (**b**) Toxicity of ethanol determined by XTT assay. (**c**) Dry weight estimation to identify the optimal extraction time for fruiting bodies extracts. The same amount of fruiting bodies powder was weighed, but different amounts of substances were dissolved in the ethanol extraction solution (40%). (**d**) Comparison of Reishi to some common antioxidants [11].

Figure 1a shows the results of the DPPH assay. The antioxidative potential of Reishi, expressed as percent radical scavenging activity (Equation (1), Section 2.1.3), was observed for three different Sunday Natural extracts (Sunday Natural 1, 2, and 3) and one Nature’s Finest extract over three months and compared to that of vitamin C. The variation in oxidative potential within the analyses was large, as indicated in the standard deviations, but comparable to those of the vitamin C control. Considering that the vitamin C samples were prepared freshly with a pure substance, the standard deviation in the fruiting bodies extracts over this long period is not that large, indicating an issue with the DPPH assay’s reproducibility, which can also be seen in our previous study by Imb et al. [11].

Figure 1b shows a solvent toxicity evaluation indicating the minor toxicity of ethanol up to approximately 2.5%. Ethanol extracts were prepared at a final concentration of one percent for treating cells to avoid the toxic effects.

The transition of molecules in solution was observed by performing dry weight estimations of the different Reishi extracts. We wanted to know how long it takes until all the soluble substances pass over to the liquid (ethanolic) phase. The Sunday Natural extracts showed better solubility; more substance was transferred from the powder into the ethanolic solution. Nature’s Finest had a lower amount of the dry substance, although the same amount was weighed (Figure 1c). Additionally, Reishi was compared to other antioxidants as can be seen in Figure 1d.

#### 3.1.2. Celldescription

An HDF primary cell line was obtained from ATCC and used in previous studies by the authors [10,11]. All experiments were performed at least 50% before the tested end of growth potential. This cell line, which had quite a low doubling rate, was passaged for about 40 population doublings. The primary cell line did not reach replicative senescence in the time that it was cultivated.

#### 3.1.3. Definition of Senescent State

**Figure 2:**
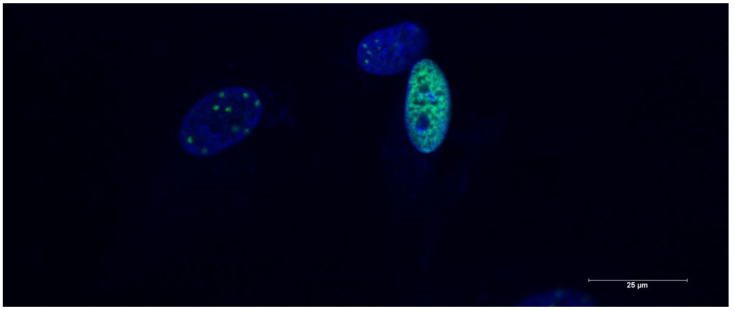
Image of etoposide-treated human dermal fibroblast cells stained for үH2AX. The green dots indicate positively stained foci due to DNA strand-break formation. Blue nuclei were stained with DAPI (4′,6-diamidino-2-phenylindole).

**Figure 3:**
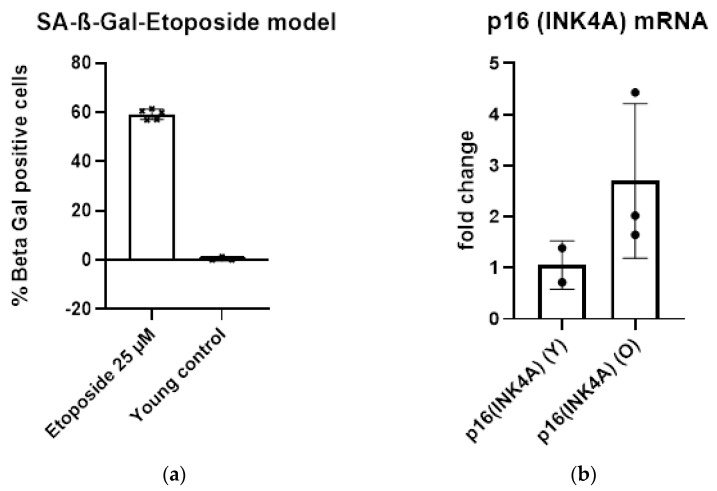
Verification of the establishment of the senescent state in human dermal fibroblasts. (**a**) Senescence-associated β-galactosidase (SA-ß-Gal) positive cells in low population doubling cells (young control) and etoposide-treated cells; (**b**) p16^INK4A^ qPCR results comparing young control (Y) and cells 14 days after exposure to etoposide (O).

Etoposide-induced senescence was proven by үH2AX staining (Figure 2) and SA-ß-Gal (Figure 3a), as well as by qPCR of the p16^INK4A^ mRNA (Figure 3b). Furthermore, etoposide-induced senescence in this cell line has been confirmed in previous publications [10,11].

#### 3.1.4. Expression of Relevant Genes in Etoposide-Induced Senescent Cells

**Figure 4:**
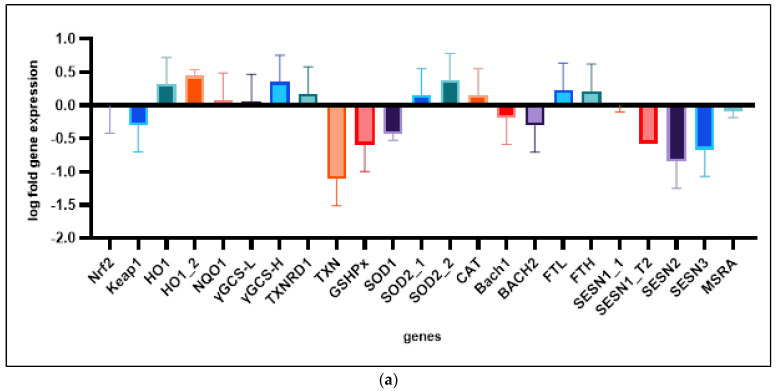

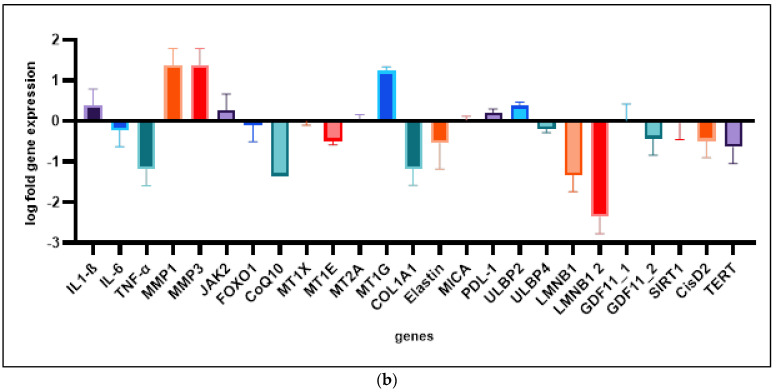
Gene expression in etoposide-induced senescent human dermal fibroblasts: (**a**) relevant genes of the antioxidative stress response; (**b**) genes of the SASP and extracellular matrix and markers of cellular senescence.

Various genes involved in aging, antioxidative metabolism (Figure 4a), and SASP (Figure 4b) were tested for their expression in etoposide-induced senescent cells. Two samples with low population doublings and two etoposide-induced senescence samples were analyzed.

The relevant genes were analyzed for an exact definition of the senescent state of HDF. Furthermore, we found out which genes are relevant for our following gene expression studies. We evaluated different primers (sequences are listed in qPCR Execution and Evaluation Section). Some of the regulations could not be confirmed in our time-course experiments, perhaps due to the higher etoposide concentration in this experiment (50 µM).

#### 3.1.5. Growth Curves

**Figure 5:**
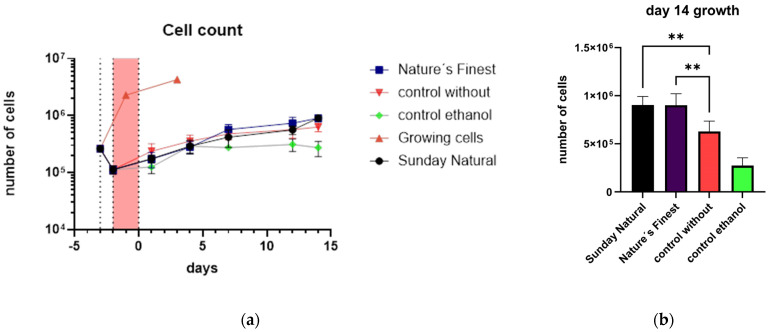
Growth curves of human dermal fibroblasts (**a**) in logarithmic scale. Growing cells are cells that were not etoposide-treated. These cells continued to grow until they became dense. Other treatments were etoposide-treated and a clear decrease in growth rate was observed dotted line shows the timepoint of seeding of cells and the red shaded area indicates the etoposide treatement; (**b**) day 14 of treatment. Columns represent different treatments. Significant differences between the Reishi (Nature’s Finest and Sunday Natural) treatments and the control without Reishi (water instead of Reishi extract) were observed. The solvent treatment (ethanol control; ethanol instead of Reishi extract) shows an even lower growth rate. Significances of means: <0.01 (**).

Treatments were performed over 17 days. Cells were seeded, and after adherence to the T-flask for one day, they were treated with 25 µM etoposide for 48 h (the red-shaded area in the graphs indicates when treatment occurred). This treatment was performed for all controls and the Reishi-treated samples except for the growing cells control. The controls were prepared as follows: the control without extract (triangles) was etoposide-treated with no extract, but with water instead at a non-toxic concentration determined in a former publication [11]; the control with ethanol (diamonds) was also etoposide-treated, but the extract was replaced by ethanol in the same amount (this control was only analyzed once and served as the solvent control); Sunday Natural (circles); and Nature’s Finest (cubes) (Figure 5a). Figure 5a shows, furthermore, that etoposide lowered the growth rate, but the cells were still alive, indicating the successful induction of cellular senescence in most of the cells. The figure is in logarithmic scale to show the growth of non-etoposide-treated cells, which continued until they became dense in the T-flask. These experiments were conducted twice with duplicate counts. What can be seen is that Reishi is somehow beneficial for the growth of HDFs under these harsh conditions of etoposide treatment (Figure 5b). In all cases, growth was not absolutely inhibited, but as seen in the growing cells group, the non-etoposide-treated cells grew to massively higher cell numbers than the treated cells.

**Figure 6:**
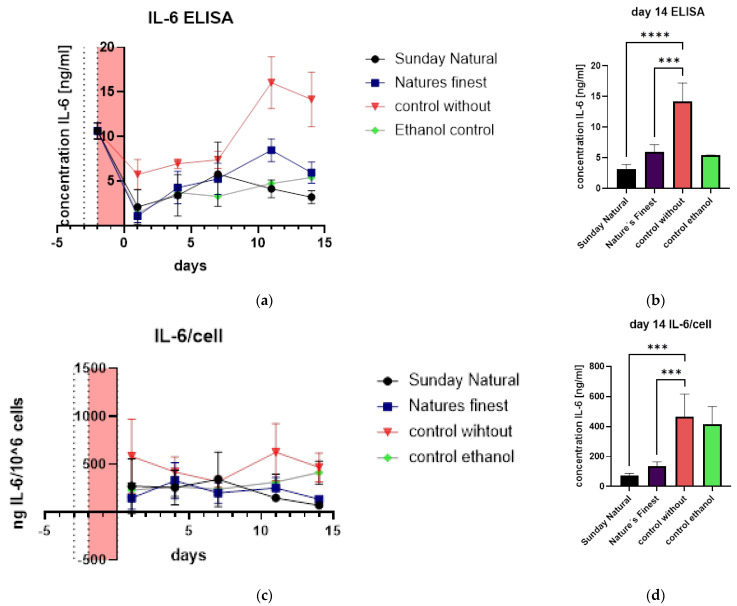
Senescence-associated secretory phenotype (**a**) IL-6 expression on protein levels uncovers a slight expression of IL-6 in all groups but a clear lesser expression in Reishi-treated groups. (**b**) Day 14 of treatment shows significant differences in IL-6 expression between the control and the Reishi-treated groups. (**c**) When IL-6 is related to cell numbers, the senomorphic effect can also be seen and in the bar graph (**d**), it is even more visible. Significances of mean: <0.001 (***); <0.0001 (****).

The growth curves were produced by counting the harvested cells after each timepoint. Gene expression and interleukin-6 (IL-6) protein data were obtained in the same experiments (Figure 6). Treatment groups were as follows: Reishi extracts were used for cell treatment after exposure to etoposide; the control without Reishi is an etoposide-treated group in which water replaced the extract; the control with ethanol is the solvent control group where ethanol was used instead of extract.

### 3.2. Ganoderma Lucidum and IL-6 Expression

Regarding SASP, we analyzed IL-6, a prominent protein that is part of the SASP, for its expression in fibroblasts, by ELISA. IL-6 was not highly expressed in the control without Reishi group, but a slight increase was observed (Figure 6c). Compared to this slight increase, we saw significantly less IL-6 protein in the Reishi groups (Sunday Natural and Nature’s Finest), indicating the senomorphic properties of these extracts (Figure 6a,b).

Differences are confirmed when relating IL-6 concentrations to cell numbers (Figure 6c,d). In general, IL-6 was not highly induced in this treatment series. Nevertheless, a decrease in IL-6 was observed in the Reishi-treated samples with both extracts.

### 3.3. Senolyotic Properties of Reishi

**Figure 7:**
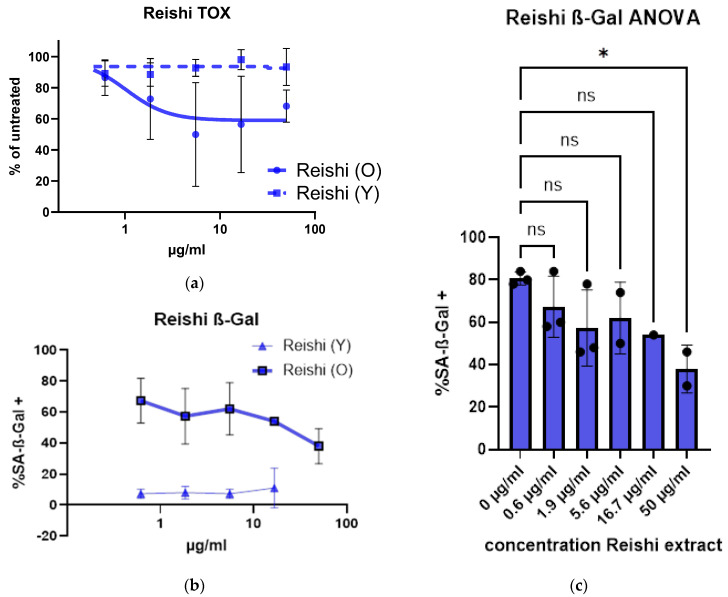
Senolytic properties of Reishi extract: (**a**) toxicity study of ethanolic Reishi extract, with etoposide-treated cells (O) showing a response in the Presto Blue assay with an IC_50_ value of 38.5 µg/mL; (**b**) decreased senescence in human dermal fibroblasts by Reishi treatment was observed in the SA-ß-Gal assay; (**c**) ANOVA of data of (**b**) * indicates a significance level of 95% (data previously published [11]). Significances of means: <0.05 (*).

Figure 7 illustrates a senolytic effect as determined by the Presto Blue assay. Reishi seems non-toxic to fibroblast cells with low population doublings in the tested concentrations up to 50 µg/mL. With a high standard deviation, there was a hint of senolysis in etoposide-treated (48 h pulse of etoposide and an establishment duration of senescence of about 14 days) old cells measured in the same way as the young cells (data previously published [11]).

In Figure 7b, cells were treated with different concentrations of Reishi extract. SA-ß-Gal staining showed that the percentage of senescent cells decreased by treatment. When performing an ANOVA (Figure 7c), even the highest concentration is significantly decreased (50 µg/mL) (data previously published [11]).

### 3.4. Analysis of Gene Expression

The mitochondria-associated gene expression (SOD2 and CDGSH iron–sulfur domain-containing protein 2 (CISD2)) was not affected by etoposide treatment or treatment with Reishi extracts.

**Figure 8:**
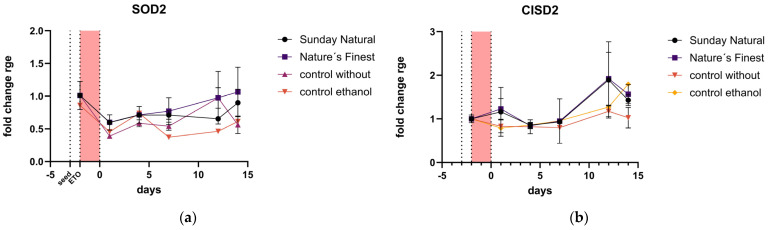
Mitochondria-associated gene expression: (**a**) the dotted line labeled with “seed” indicates the beginning of the experiment where cells were seeded in the T-flask and ETO (etoposide) at the beginning of the red area indicates the etoposide treatment for 48 h. SOD2, if any, was about half downregulated by etoposide treatment, but there was no difference in the extract or control treatments; (**b**) CISD2 was also not affected by the treatment, and the pattern is quite equal between groups.

Mitochondrial SOD2 was not regulated by Reishi extracts compared to the control (Figure 8a), and there was a slight downregulation of this gene by etoposide treatment. CISD2 encodes a protein found in the outer membrane of mitochondria and is also not affected by etoposide treatment (Figure 8b).

**Figure 9:**
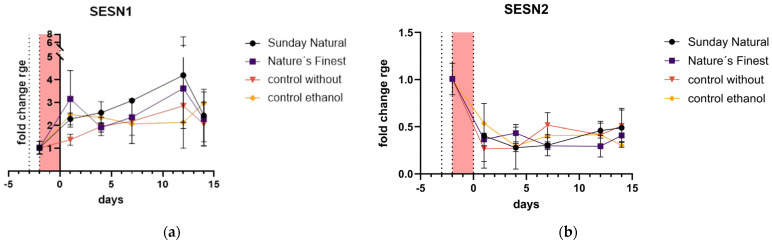
Sestrin expression: (**a**) Sestrin1 (SESN1) expression was induced in all treatments and is comparably higher in the Reishi-treated cells than in the non-extract-treated control; (**b**) Sestrin2 (SESN2) expression was downregulated in all treatment groups. There were no differences between the groups.

Sestrins 1 and 2 were regulated in different directions. Sestrin1 (SESN1) was upregulated in all conditions tested. The immediate response one day after etoposide treatment with the two sources of Reishi was higher than the response of the non-extract control. The ethanol control showed a similar pattern to the Reishi ethanolic extracts on this day. Over the whole timeframe of 14 days post-etoposide treatment, the Reishi extracts induced more or equal SESN1 than the non-extract-treated control (Figure 9a). Sestrin2 (SESN2), if any, was downregulated in all treatments and over the 14 days (Figure 9b).

**Figure 10:**
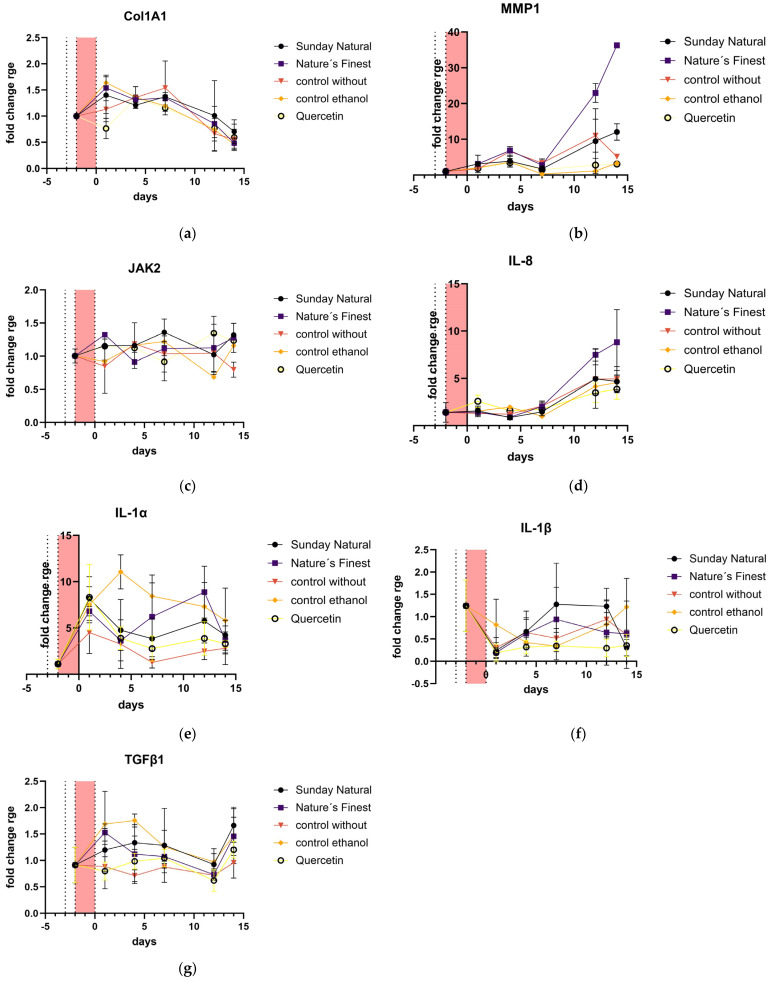
Extracellular matrix-associated gene expression: an additional senomorphic control (quercetin) was included in all gene expression graphs: (**a**) ColA1 was moderately upregulated in all treatment groups at the beginning of the treatment period but downregulated at day 14 in all treatment and control conditions; (**b**) MMP1 was massively induced by etoposide treatment, while there was no difference between the control without extract and the Reishi-treated samples; however, MMP1 in the ethanol control was upregulated after day 14. The axis is in logarithmic scale due to the large differences in expression; (**c**) JAK2 expression was not affected by all treatments. (**d**) IL-8 shows the same pattern as MMP1 and shows differences in the two Reishi extracts; for (**e**) IL-1α and (**f**) IL-1β, compared to their expression with quercetin, both genes are slightly upregulated. (**g**) TGFβ1 was not significantly affected by different treatments.

Extracellular matrix-related genes and genes of the SASP ColA1 were upregulated in the first reaction to etoposide treatment, but as cellular senescence was established with time, on days 10 to 14, it was downregulated in all treatments and controls, and there was no difference between the samples and controls (Figure 10a). Conversely, MMP1 was massively upregulated (Figure 10b). MMP1 expression in the Nature’s Finest extract-treated samples increased about 36-fold after day 14 (Figure 10b) despite lesser solubility in our solubility studies (Figure 1c). JAK2 (Figure 10c) expression was not affected by the treatments. A senomorphic positive control (quercetin) was included in the SASP gene expression studies; this known senomorpic substance shows decreased MMP1 levels. IL-8, a main gene of the SASP, is regulated in the same way as MMP1, and Nature’s Finest also shows a massive induction of IL-8. The other preparation shows similar IL-8 induction as the control groups. The IL-1α (Figure 10e) expression of Natre’s Finest is also increased at the timepoint of 12 d; the Sunday Natural preparation behaves similarly to the controls, except for the ethanol control, which is massively upregulated on day 4. IL-1β (Figure 10f) had very low expression generally, but quercetin caused lower expression than the Reishi extracts did, indicating, again, that there was no senomorphic effect from the Reishi extracts. The growth factor TGFβ1 (Figure 10g) was not highly influenced by either Reishi extracts, similarly to the IL-1α gene showing that ethanol increased its expression at day 4.

Antioxidant defense and cytoprotection in HDF were upregulated. Nrf2 is the master regulator of the antioxidant response. When activated, it translocates to the nucleus and induces the expression of heme oxygenase-1 (HO-1), gamma-glutamylcysteine synthetase light subunit, also known as GCLM (γGCS-L), and NAD(P)H quinone oxidoreductase 1 (NQO1), among many other cytoprotective genes. HO-1 catalyzes the breakdown of heme into biliverdin, iron, and carbon monoxide, which have antioxidant and anti-inflammatory properties [36]. γGCS-L is involved in glutathione synthesis, a major cellular antioxidant. NQO1 catalyzes the two-electron reduction of quinones, preventing the formation of ROS [37].

**Figure 11:**
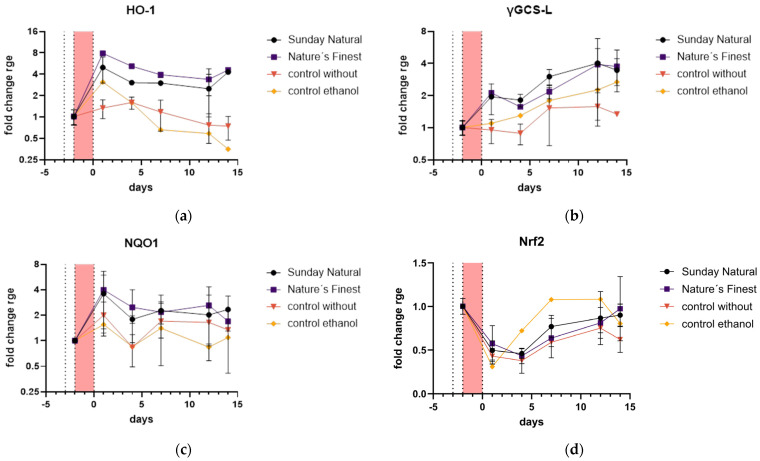
Antioxidant defense and cytoprotection gene expression: (**a**) HO-1 was upregulated in the Reishi-treated groups compared to the control without extract. The expression increased rapidly and stayed high over the whole test period. (**b**) γGCS-L was also upregulated but increased for the whole period. The expression for the Reishi-treated samples was about double that of the control group. (**c**) NQO1 had a similar pattern to HO-1 but was not that differentially expressed compared to the control group. (**d**) Nrf2 expression was comparable between the control and the Reishi-treated groups and a little downregulated by etoposide treatment but returned to normal expression after 14 days.

HO-1 was upregulated compared to the non-extract control (Figure 11a). In the beginning phase of the treatment, there was a nearly eight-fold upregulation of HO-1 in both Reishi extract groups compared to a slight upregulation in the control group. The high upregulation of HO-1 remained for the whole period of 14 days post-etoposide treatment.

A similar trend was observed in the γGCS-L expression pattern, but the expression increased continuously over the 14 days. There was also a big difference in both Reishi values and the no-extract control, suggesting an upregulation of antioxidant defense and cytoprotection (Figure 11b).

The NQO1 expression in the Reishi extract samples was double that of the no-extract control over the whole period of 14 days, indicating a constant upregulation of this gene (Figure 11c). Nrf2 was downregulated at the beginning of the 14 days but returned to normal before the end of the investigation (Figure 11d).

**Figure 12:**
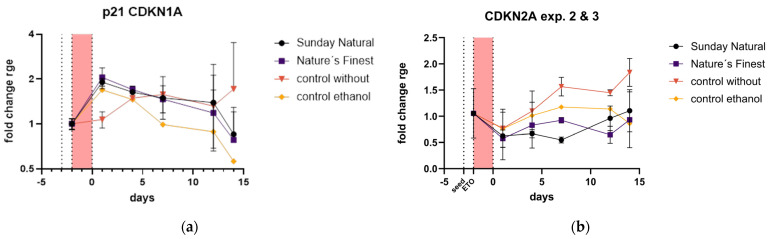
Cell cycle and senescence-associated genes: (**a**) p21^CDKN1A^ upregulation was delayed in the control without treatment group compared to the Reishi extract-treated groups; (**b**) p16^CDKN2A^ was downregulated or not regulated by Reishi treatment.

Reishi downregulated p16^CDKN2A^ expression and initiated the early upregulation of p21^CDKN2A^. Initially, p21^CDKN2A^ was upregulated one day after Reishi treatment, about twice that of the control without extracts. Later, the pattern aligns with the control and stays nearly the same (Figure 12a).

Most importantly, p16 ^CDKN2A^ was not upregulated and stayed near zero compared to the slowly increasing p16 expression in the control without extract. So, despite the clear initial upregulation of p21^CDKN2A^, there was no later detection of the most important marker of cellular senescence, namely p16^CDKN2A^ (Figure 12b).

## 4. Discussion

*Ganoderma lucidum*, commonly known as the Reishi fruiting bodies, has garnered significant attention for its potential anti-aging properties and ability to combat cellular senescence and oxidative stress. This medicinal fungus contains various bioactive com-pounds, including polysaccharides, triterpenes, and phenolic compounds, contributing to its diverse therapeutic effects. Research has demonstrated that Reishi extracts can effectively promote resistance to oxidative stress and extend the lifespan in model organisms such as *Caenorhabditis elegans*. The fruiting bodies’ antioxidant properties help protect cells against damage caused by ROS and other free radicals, which are major contributors to the aging process. *Ganoderma lucidum* activates multiple signaling pathways involved in the stress response and longevity. For instance, it can modulate the diet restriction and mTOR/S6K signaling pathways to protect against oxidative insults. Additionally, Reishi extracts influence the germline signaling pathway, which plays a crucial role in regulating lifespan [38].

At the cellular level, Reishi and its compounds, such as ganoderic acid D, have demonstrated the ability to inhibit senescence in various cell types, including stem cells. These effects are mediated by activating antioxidant defense mechanisms, such as the protein kinase RNA-like endoplasmic reticulum kinase/Nrf2 signaling pathway, which enhances the expression of cytoprotective genes. Furthermore, *Ganoderma lucidum* has shown promise in mitigating age-related oxidative damage in animal models. It can enhance the activity of antioxidant enzymes like superoxide dismutase (SOD) and glutathione peroxidase while reducing the levels of oxidative stress markers such as malondialdehyde [39,40].

By defining the reagents, we found that Reishi ethanolic extracts did not have a high antioxidative potential. The antioxidative potential stayed nearly constant over the time we treated the cells. High standard deviations, also seen in the vitamin C standard tests, indicate a big variation between DPPH assays.

Antioxidants have complex effects on cellular senescence, depending on their dose, type, and the cellular context. While moderate doses of antioxidants can protect against oxidative stress and potentially reduce senescence, high doses of synthetic antioxidants can paradoxically induce premature senescence in proliferating cells [41]. This effect is particularly pronounced when antioxidants are applied to cells maintaining physiological levels of reactive oxygen species.

Oxidative stress plays a crucial role in cellular senescence and aging. Antioxidant enzymes like superoxide dismutase (SOD) and glutathione (GSH) are important in combating oxidative stress, but their levels and activities can change with age [42]. This highlights the complex relationship between antioxidants, oxidative stress, and cellular senescence in the aging process. Overall, while antioxidants show promise in modulating senescence-related processes, their effects are nuanced and context-dependent. Careful consideration of dosage, timing, and the cellular environment is crucial when exploring antioxidant interventions for senescence-related conditions.

Nevertheless, a rough estimate of antioxidative potential could be made, and this potential was maintained over a long time (three months) when keeping the extracts cool and dark. During the extraction period of 40 days, we observed the dissolving of substances in 40% ethanol. This showed that more substances were dissolved in the Sunday Natural group considered in the experiments. As we do not know the active ingredients, we focused on this summative parameter for measuring the ability of 40% ethanol to extract substances out of the powdered fruiting bodies preparations.

The common hallmarks of senescence are expressed in etoposide treatment, as we reported in previous publications [10,11]. үH2AX staining was positive for foci due to strand-break formation in the nuclei of HDF cells. SA-ß-Gal staining showed increased staining in the etoposide treatment group (25 µM). In these experiments, we raised the concentration of etoposide treatment to 50 µM, which is double the concentration in previous experiments [10,11]. The expression of p16^CDKN1A^, measured by the qPCR of mRNA levels in HDF cells, increased with etoposide treatment.

We tested many genes (using primer sequences from the literature) representing a detailed gene pattern for etoposide treatment. Unfortunately, IL-6 expression was not really affected by 50 µM etoposide treatment. The other genes mostly show similar expression as proposed in the literature. To our knowledge, a qPCR gene expression analysis of etoposide-induced senescence in this level of detail has not been performed by others before.

Massive exposure to chemo-therapeutic etoposide (50 µM) led to growth arrest. A little residual growth was observed as not all cells will ever be senesced. Interestingly, both Reishi groups showed increased growth compared to the water-treated control, indicating a moderate alleviation of etoposide’s damaging effects.

Despite the low expression of IL-6 in these treatment conditions, we observed a decrease in this inflammatory cytokine. A reduction in IL-6 was observed also when IL-6 concentrations were related to cell numbers. In a similar study, IL-6, IL-1β, and IL-1α were markedly reduced in the triterpenoid complex of *Ganoderma lucidum*, as evidenced by qPCR. We confirmed the protein level findings by Abdelmoaty et al. [43]. The decrease in IL-6 protein is significant but not very prominent; as mentioned, IL-6 is normally very highly expressed which is not the case in these treatments, and because of that, we analyzed additional genes of the SASP which point in another direction discussed later in this text.

The results suggest that Reishi extract exhibits senolytic properties, as evidenced by the decrease in senescent cells observed in the SA-β-Gal assay. This effect was concentration-dependent and statistically significant at the highest concentration. These findings indicate that Reishi could target and eliminate senescent cells, which may be beneficial in combating skin aging. Additionally, these extracts have a more toxic effect on old cells (etoposide treated) than on cells with low population doublings. Again, referring to Abdelmoaty et al. [43], we, in contrast, used a primary HDF cell line, which should be quite untransformed in comparison to cancer cells. Senescence in somatic cells plays a role in cancer progression. Additionally, Abdelmoaty et al. [43] induced senescence for five days only, which is not an optimal duration for establishing cellular senescence, to our knowledge.

This study on the triterpenoid complex from *Ganoderma lucidum* also found a senolytic effect against senescent hepatocellular carcinoma cells. They found that the triterpenoid complex from *Ganoderma lucidum* had a senolytic effect, which could selectively eliminate adriamycin-induced senescence in hepatocellular carcinoma cells via caspase-dependent and mitochondrial pathway-mediated apoptosis and reduce the levels of senescence markers [43]. Adriamycin (doxorubicin) belongs to the group of intercalants. Its effect is based on intercalation into the DNA. Doxorubicin acts as an intercalant on the planar compounds in DNA and RNA. DNA synthesis is disturbed, and, like etoposide, topoisomerase II is inhibited, and radical formation occurs. This study and Abdelmoaty et al. [43] show comparable results. However, this study goes into more detail, and we offer interesting new results regarding gene expression.

The expression of mitochondria-associated genes (SOD2 and CISD2) was not significantly affected by Reishi treatment. This indicates that the observed effects of Reishi on antioxidant defense may be primarily mediated through non-mitochondrial antioxidative pathways.

SESNs are cysteine sulfinyl reductases that play critical roles in oxidant defense regulation. SESN1 and SESN2 share some common functions; SESN2 appears to have a broader range of effects and is more extensively studied in various physiological and pathological contexts [44,45]. SESN1 seems more specifically associated with the genotoxic stress response, while SESN2 has more diverse roles in cellular homeostasis and stress adaptation. In some contexts, SESN1 and SESN2 may have overlapping functions and could compensate for each other, but this is not always the case and depends on the specific cellular context and stress conditions. While SESN1 is primarily regulated by p53 and responds to genotoxic stress in a p53-dependent manner, SESN2 can be regulated by multiple factors, including p53, Nrf2, ATF4, C/EBPβ, JNK/c-Jun, AP-1, and HIF1 [46]. The upregulation of SESN1 might indicate that etoposide is inducing sharply distinguished damage to HDF cells, inducing strand breaks and p53. Without additional oxidative stress or starving, SESN2 might not be induced. Additionally, if SESN2 fails to be appropriately upregulated in senescent cells, it could lead to reduced autophagy activation, as published recently [43]. Both effects are true for all treatments and seem to be due to the etoposide treatment itself which was performed in all treatments displayed.

MMP-1 degrades ColA1, which, in turn, can affect the expression of ColA1 genes. Fibroblasts cultured in MMP-1-fragmented collagen lattices showed reduced collagen production [47]. Our experiments show that MMP-1 (Collagenase-1) expression is generally inversely related to ColA1 expression [48]. Unfortunately, Reishi extract cannot alleviate this effect of skin aging. The difference in MMP1 between the two Reishi extracts could be due to differences in preparation; one of them seems to have inflammatory ingredients not specified in detail.

Taking a deeper look into the SASP and related gene expression shows that one of these Reishi extracts is also inducing an inflammatory factor, namely IL-8, which is massively upregulated by this preparation. The second preparation shows similar upregulation to the non-extract-treated control and at least no negative effect. Here, we included a quercetin control, which is a known senomorphic compound. Neither in IL-1α nor in IL-1β expression do the Reishi extracts show any senomorphic properties like those of quercetin.

JAK2 plays a crucial role in the activation and profibrotic behavior of HDFs [49]. It is involved in collagen synthesis, the response to growth factors and cytokines, and the signal transduction pathways that contribute to fibrosis. Cytokine stimulation induces the phosphorylation of JAK2 at multiple residues, which regulates its activity either positively or negatively [50]. Generally, it is regulated post-translationally, so having no change in expression does not mean it is not involved in this process.

This study demonstrated a significant upregulation of key antioxidant and cytoprotective genes in the Reishi-treated cells compared to the controls. Specifically, HO-1 showed a rapid and sustained upregulation in the Reishi-treated groups, indicating an enhanced antioxidant response. γGCS-L expression increased continuously over the 14-day period, suggesting improved glutathione synthesis capacity. Furthermore, γGCS-L was higher in the Reishi-treated samples. NQO1 expression was consistently higher in the Reishi-treated cells, further supporting an enhanced antioxidant defense mechanism. These findings suggest that Reishi extracts may help protect dermal fibroblasts against oxidative stress, which is a key factor in cellular senescence and skin aging.

Interestingly, our study found that Reishi treatment affected the expression of key senescence markers. Most interesting, p16^CDKN2A^ expression, a critical marker of cellular senescence, was suppressed in the Reishi-treated cells compared to the controls. This suggests that Reishi may help prevent or delay the onset of senescence in dermal fibroblasts. Additionally, p21^CDKN1A^ showed an initial upregulation in the Reishi-treated cells, followed by a return to control levels. This transient increase in p21^CDKN1A^ might indicate a temporary cell cycle arrest, possibly allowing cellular repair mechanisms to take effect.

In summary, we have observed and reported the very interesting effects of Reishi (*Ganoderma lucidum*), pointing traditional medicine in a direction where it is able to alleviate signs of aging. It would be very interesting to see if supplementing with Reishi would affect signs of aging in humans.

Many traditional medicines have long histories of use in human populations, often spanning centuries. This provides valuable long-term safety data that are difficult to obtain for newly developed drugs [51]. Since many traditional medicines are already approved as dietary supplements or foods, they may face fewer regulatory restrictions than novel pharmaceutical compounds. This could accelerate the research process, making it easier to conduct clinical studies. Traditional medicines often have higher levels of acceptance among older populations due to cultural familiarity. This could aid in recruitment for clinical trials and improve compliance, which is crucial for studying the long-term effects of aging [52]. Traditional medicines are often more affordable than newly developed drugs. This could be beneficial for conducting large-scale, long-term clinical studies needed to assess the effects on aging processes. Some traditional medicines may already have data on their effects in age-related conditions. The current research could provide a foundation for investigating Reishi’s potential senolytic properties, potentially streamlining the research process.

However, it is important to note that traditional medicines still require rigorous scientific evaluation to establish their efficacy and safety as senolytics. Reishi does not show any senomorphic effect in this study; on the contrary, one Reishi extract showed an increase in the SASP factor IL-8 and MMP1 which confirms the problematic fact that compositions of different preparations of the same natural product can have a deep impact on the effect of these medicines. While they may offer some advantages in the research process, they should be held to the same standards of evidence as other interventions. As also seen in this study, standardization of traditional medicine formulations can be challenging and needs to be addressed in clinical studies.

## 5. Conclusions

The results suggest that *Ganoderma lucidum* extract may have potential to modulate cellular senescence in HDFs, primarily through enhanced antioxidant defense and cytoprotection mechanisms. The suppression of p16 expression and the senolytic properties observed are particularly promising. However, the lack of an effect on ECM-related genes suggests that Reishi’s benefits may be limited to certain aspects of cellular aging. Further research is needed to fully elucidate the mechanisms of action and potential applications of Reishi extract in combating skin aging.

## Figures and Tables

**Table 1:** Steps for the thermal cycler.

	Priming	Reverse Transcription	Inactivation	Hold
Temperature [°C]	25	46	95	4
Duration [min]	5	20	1	10

**Table 2:** qPCR temperature profile.

	Polymerase Activation	Denaturation	Annealing
Temperature [°C]	95	95	60
Duration [s]	30	5	30
Cycles	1	40	

## Data Availability

Data is contained within the article.
